# The mitogenome of *Triatoma brasiliensis brasiliensis* (Hemiptera: Reduviidae), the main Chagas disease vector in the semi-arid region of northeastern Brazil

**DOI:** 10.1186/s13071-025-06769-0

**Published:** 2025-04-04

**Authors:** Carlos E. Almeida, Lifeng Du, Jingwen Wang, Dayane Pires-Silva, Elaine Folly-Ramos, Myrian Harry, Cleber Galvão

**Affiliations:** 1 Laboratório Nacional e Internacional de Referência em Taxonomia de Triatomíneos, Instituto Oswaldo Cruz-FIOCRUZ, Av. Brasil 4365, Pav. Rocha Lima, Manguinhos, Rio de Janeiro, RJ CEP 21040-900 Brazil; 2 Ministry of Education Key Laboratory of Contemporary Anthropology, School of Life Sciences, Fudan University, Shanghai, 200438 People’s Republic of China; 3 Universidade de Campinas, São Paulo, Brazil; 4 Universidade Federal da Paraíba-UFPB Campus IV-Litoral Norte, Rio Tinto, Brazil; 5 Paris-Saclay University, Gif-sur-Yvette, France

**Keywords:** Triatominae, Vector-borne diseases, Mitochondrial DNA sequencing, Insect genomics

## Abstract

**Background:**

*Triatoma brasiliensis brasiliensis* is the primary vector of Chagas disease in Brazil's semi-arid regions, exhibiting adaptability to various environments, including domestic and peridomestic. Despite its significance, comprehensive genomic data for this subspecies remain limited.

**Methods:**

We assembled the complete mitochondrial genome of *T. b. brasiliensis* using a combination of Illumina and Sanger sequencing technologies, the latter being necessary to obtain the control region with eight primers designed in this study. The mitogenome was annotated to identify gene content and organization. Phylogenetic relationships were inferred using conserved blocks of 13 protein-coding genes and 22 transfer RNA genes. For this analysis, 18 representative triatomines with near-complete mitogenomes were selected, and phylogenetic reconstruction was performed using the maximum ikelihood method.

**Results:**

The complete mitogenome spans 16,575 base pairs and includes 13 protein-coding genes, 22 transfer RNA genes, and two ribosomal RNA genes, consistent with the typical structure of insect mitochondrial genomes. The control region exhibited tandem and inverted repeats arranged in blocks, as observed for other Reduviidae. Given the limited availability of mitogenomes, our phylogenetic analysis provided statistical support for *T. b. brasiliensis* as a sister taxon to *Triatoma infestans*, forming a well-supported clade that is sister to *Triatoma vitticeps*.

**Conclusions:**

The availability of this mitogenome provides insights into the systematics, biology, and genomics of triatomine species while also enhancing our understanding of their evolutionary relationships. However, the limited number of available mitogenomes, particularly for South American *Triatoma* species, underscores the need for further sequencing efforts to improve phylogenetic resolution and support comparative genomic studies.

**Graphical Abstract:**

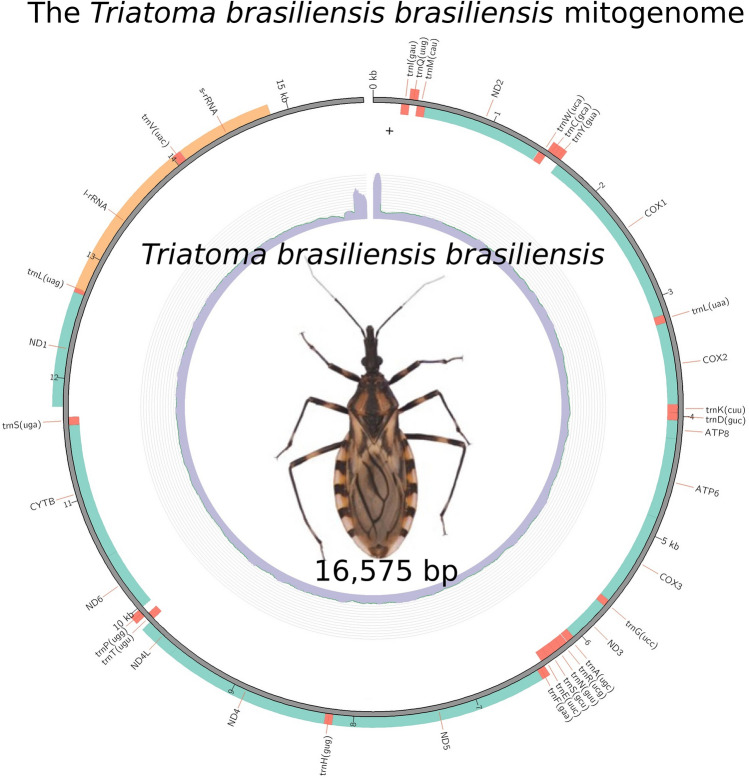

**Supplementary Information:**

The online version contains supplementary material available at 10.1186/s13071-025-06769-0.

## Background

Currently, 158 species of triatomines (Hemiptera: Reduviidae) are recognized, distributed across 18 genera and 5 tribes. The most recently described species, *Triatoma atrata* and *T. picta*, were identified in 2023 [1]. In Brazil, synanthropic species such as *Triatoma brasiliensis*, *T. infestans*, *T. pseudomaculata*, *T. sordida*, and *Panstrongylus megistus* are major public health concerns [2] due to their critical roles as vectors of Chagas disease. In the semi-arid regions of Brazil, *T. brasiliensis brasiliensis* is the primary vector of Chagas disease, exhibiting remarkable adaptability to domestic, peridomestic, and sylvatic environments [3, 4]. Its natural habitats, primarily rocky outcrops [5], are inaccessible to conventional vector control methods, leading to frequent and rapid reinfestation of domiciles following insecticide applications. This poses a significant challenge to efforts aimed at mitigating Chagas disease transmission [3, 6, 7]. This species is the nominal taxon of a complex that includes seven species: *Triatoma brasiliensis, T. bahiensis, T. juazeirensis, T. lenti, T. melanica, T. petrocchiae*, and *T. sherlocki* [8]. Within this complex, *Triatoma brasiliensis* is further divided into two subspecies: *T. b. brasiliensis* and *T. b. macromelasoma* [9]. Members of this group display distinct morphological traits and varying degrees of epidemiological relevance [9, 10]. Among these, *T. b. brasiliensis* is the most adapted to peridomestic and domestic environments. In these settings, it frequently exhibits high rates of natural *Trypanosoma cruzi* infection, further amplifying its role as a critical vector in Chagas disease transmission [11, 12].

Advances in the systematics of Triatominae have primarily been driven by Sanger sequencing [13–15], although recent studies have increasingly employed phylogenomics to provide deeper evolutionary insights [16–18]. Despite substantial progress in sequencing technologies and analytical approaches, the number of fully sequenced and annotated mitochondrial genomes for Triatominae species remains limited, especially considering their diversity and epidemiological importance [18–23]. In this study, we present the assembled and annotated mitogenome of *T. b. brasiliensis*, contributing to the growing genomic resources for exploring genetic diversity and advancing systematics within the Triatominae.

## Methods

A *T. b. brasiliensis* sample collected in Currais Novos, Rio Grande do Norte, Brazil (6°15′39″S, 36°30′54″W), was used for this analysis. Total nucleic acids were extracted from midgut tissue using the Qiagen extraction kit (Promega®) according to the manufacturer’s instructions. DNA quantification and integrity were assessed using the Qubit 3.0 High Sensitivity DNA Assay (ThermoFisher, USA). High-quality DNA was used to prepare libraries following the Illumina TruSeq Nano DNA Library Kit protocol (Seoul, Korea). Sequencing was performed in Macrogen on the Illumina NovaSeq 6000 platform, generating more than 18 million paired-end reads of approximately 150 bp with a GC content of 34% after trimming, consistent with expectations for this dataset.

The mitochondrial genome was assembled using Mitoz v3.6 [24] and SPAdes v3.15.2 [25], with results cross-checked for consistency. Most regions demonstrated coverage exceeding 1000×; however, coverage dropped significantly after position 15,000 bp. To recover the control region, which was not entirely obtained through Illumina sequencing, a set of eight primers (MT-F1: CCTACAAAACCGCATGTTCA, MT-R1: TTTTGTTATTGGGGCTTGGC, MT-F2: CACTAACCCTTCAACGACAA, MT-R2: CCCTTTTAAAACGGGGATCG, MT-F3: AGTTAGAATTGACGCTCAG, MT-R3: CCTATTTATCAGGCACCTT, MT-F4: CATACCCGGATAGGATTAG, MT-R4: CTTGGGATCTGAGAACAAT) was designed using Primer v5.0 [26], and the resulting sequences were integrated into the final assembly. The first pair of primers was designed based on the initial sequence output from MitoZ v3.6, providing a foundation for primer placement. Subsequent sequencing results guided the design of additional primers to cover the remaining gaps in the control region. Annotation was performed using MitoZ v3.6, and the assembly was validated through MUSCLE v3.8.1551 [27] for individual genes. The circularized final version was validated by manual inspection. To enhance alignment accuracy, the 13 protein-coding genes (PCGs) and 22 transfer RNA (tRNA) genes were aligned independently with homologous genes from other triatomine species with annotated mitogenomes [18–23]. Open reading frames (ORFs) were identified using ORFfinder (NCBI, Bethesda, MD, USA; https://www.ncbi.nlm.nih.gov/orffinder) and compared with other insect mitogenomes, including *T. infestans* [23]. Stop codon positions were also confirmed by aligning sequences with reference mitogenomes, where incomplete stop codons (T or TA) are completed through post-transcriptional polyadenylation [22, 28]. Ribosomal RNA (rRNA) annotations were extended to include adjacent tRNAs, and the 5′ ends of small rRNAs (srRNAs) were determined through comparative mitogenomic analysis. Tandem repeats (TRs) within the mitochondrial genome were detected using Tandem Repeats Finder [29]. To identify and compare tandem repeat sequences from the control region in *T. b. brasiliensis* and other triatomine species, major consensus repeat motifs (18–149 bp) were selected. A BLAST database was built using complete mitochondrial genomes from available triatomine species. *Triatoma b. brasiliensis* repeat motifs were then queried against this database using BLASTN. To detect potentially homologous repeats in control regions from other species, a relaxed filtering approach was applied: identity ≥ 85%, alignment length ≥ 40 bp, *E*-value ≤ 1e−5, and bit score ≥ 50. Matches were manually verified to confirm their location within the control region of the mitochondrial genomes.

Conserved blocks of 13 PCGs and 22 tRNAs from a set of samples, representing each species complex with available mitogenomes, were selected for analysis. Gblocks v0.91b [30] was used to refine alignments and select conserved regions (12,280 bp). Phylogenetic trees were constructed using the maximum likelihood (ML) method implemented in IQ-TREE v2.2.0 [31], choosing the best-fit substitution via ModelFinder and tree search algorithm [32, 33]. Branch support was assessed using 1000 ultrafast bootstrap (BS; UFBoot2) replicates and SH-aLRT tests with default settings. *Oncocephalus breviscutum* (NC_022816) was set as the outgroup.

## Results and discussion

The mitogenome (16,575 bp; accession code PV085522; Additional File 1) of *T. b. brasiliensis* was shorter than that of *T. infestans* (17,301 bp) but longer than that of *Triatoma mexicana* (15,699 bp) [20, 23]. It contains 37 genes, including 13 protein-coding genes (PCGs), 22 transfer RNA (tRNA) genes, and 2 ribosomal RNA (rRNA) genes. These genes are arranged in the typical insect mitochondrial gene order, oriented on the same strand, and show no major rearrangements compared to closely related species. The 13 PCGs range in length from 160 base pairs (ATP8) to 1714 base pairs (ND5). The 22 tRNA genes vary in size from 63 to 71 base pairs. The rRNA genes are located between positions 12,487 and 14,619, separated by the valine tRNA, with the large ribosomal RNA (16S rRNA) measuring 1309 base pairs and the small ribosomal RNA (12S rRNA) measuring 772 base pairs. Some protein-coding genes (ATP6 and COX3) exhibit incomplete stop codons (T or TA, with COX3 annotated to have its TAA stop codon completed by the addition of 3′ A residues to the mRNA), which are completed post-transcriptionally by the addition of 3′ poly(A) tails, a common feature in mitochondrial genome expression [34]. Functional annotation revealed near-complete conservation of start and stop codons, consistent with mitochondrial genomes of related species. However, some differences were observed. For instance, ND2 in *T. b. brasiliensis* initiates with ATC, whereas *T. infestans* uses ATT, although both codons code for isoleucine and do not affect protein functionality. Similarly, ND5 and ND6 exhibit an ATA start codon in *T. b. brasiliensis*, while *T. infestans* has GTG and ATG, respectively, which may represent species-specific mutations. Additionally, ATP6 in *T. b. brasiliensis* terminates with TAG instead of TAA as in *T. infestans*, suggesting a potential stop codon variation. Moreover, COX3 in *T. b. brasiliensis* ends with an incomplete stop codon (TTA), similar to *T. infestans* (TA), both of which require post-transcriptional polyadenylation for translation termination. In contrast, ND4, ND4L, ND3, COX1, COX2, ATP8, and CYTB exhibit full conservation of start and stop codons between both species. The tRNA genes in *T. b. brasiliensis* range in length from 62 to 70 bp, while the s-rRNA and l-rRNA genes measure 771 bp and 1308 bp, respectively, with an A + T content of 71.5%, closely resembling values observed in *T. infestans* (Table 1).

**Table 1:** Features of the annotated mitochondrial genome of *Triatoma b. brasiliensis*, detailing the genomic position, type of genetic element, and its functional role

Start	End	Length (bp)	Direction	Type	Gene name	Gene prodcut	Start/stop codon
1	66	65	+	tRNA	trnI(gau)	tRNA-Ile	–
63	131	70	−	tRNA	trnQ(uug)	tRNA-Gln	–
131	198	69	+	tRNA	trnM(cau)	tRNA-Met	–
199	1197	1000	+	CDS	ND2	NADH dehydrogenase subunit 2	ATC/TAG
1203	1268	67	+	tRNA	trnW(uca)	tRNA-Trp	–
1261	1323	64	−	tRNA	trnC(gca)	tRNA-Cys	–
1324	1388	66	−	tRNA	trnY(gua)	tRNA-Tyr	–
1390	2928	1540	+	CDS	COX1	cytochrome c oxidase subunit I	ATG/TAA
2924	2990	68	+	tRNA	trnL(uaa)	tRNA-Leu	–
2991	3689	700	+	CDS	COX2	cytochrome c oxidase subunit II	ATT/TAA
3670	3738	70	+	tRNA	trnK(cuu)	tRNA-Lys	–
3739	3802	65	+	tRNA	trnD(guc)	tRNA-Asp	–
3803	3961	160	+	CDS	ATP8	ATP synthase F0 subunit 8	ATG/TAA
3955	4660	707	+	CDS	ATP6	ATP synthase F0 subunit 6	ATG/TAG
4625	5409	786	+	CDS	COX3	cytochrome c oxidase subunit III	ATG/TTA(a)
5409	5471	64	+	tRNA	trnG(ucc)	tRNA-Gly	–
5469	5825	358	+	CDS	ND3	NADH dehydrogenase subunit 3	ATA/TAG
5825	5889	66	+	tRNA	trnA(ugc)	tRNA-Ala	–
5895	5958	65	+	tRNA	trnR(ucg)	tRNA-Arg	–
5960	6024	66	+	tRNA	trnN(guu)	tRNA-Asn	–
6024	6092	70	+	tRNA	trnS(gcu)	tRNA-Ser	–
6093	6155	64	+	tRNA	trnE(uuc)	tRNA-Glu	–
6158	6225	69	−	tRNA	trnF(gaa)	tRNA-Phe	–
6225	7937	1714	−	CDS	ND5	NADH dehydrogenase subunit 5	ATA/TAA
7938	7999	63	−	tRNA	trnH(gug)	tRNA-His	–
8001	9332	1333	−	CDS	ND4	NADH dehydrogenase subunit 4	ATG/TAG
9326	9619	295	−	CDS	ND4L	NADH dehydrogenase subunit 4L	ATG/TAA
9622	9684	64	+	tRNA	trnT(ugu)	tRNA-Thr	–
9685	9753	70	−	tRNA	trnP(ugg)	tRNA-Pro	–
9754	10,257	505	+	CDS	ND6	NADH dehydrogenase subunit 6	ATA/TAA
10,257	11,390	1135	+	CDS	CYTB	cytochrome b	ATG/TAG
11,389	11,456	69	+	tRNA	trnS(uga)	tRNA-Ser	–
11,540	12,475	937	−	CDS	ND1	NADH dehydrogenase subunit 1	ATA/TAA
12,458	12,522	66	−	tRNA	trnL(uag)	tRNA-Leu	–
12,487	13,794	1309	−	rRNA	l-rRNA	16S ribosomal RNA	–
13,776	13,845	71	−	tRNA	trnV(uac)	tRNA-Val	–
13,848	14,618	772	−	rRNA	s-rRNA	12S ribosomal RNA	–
14,619	16,575	1055	−	–	–	Control region	–

**Fig. 1:**
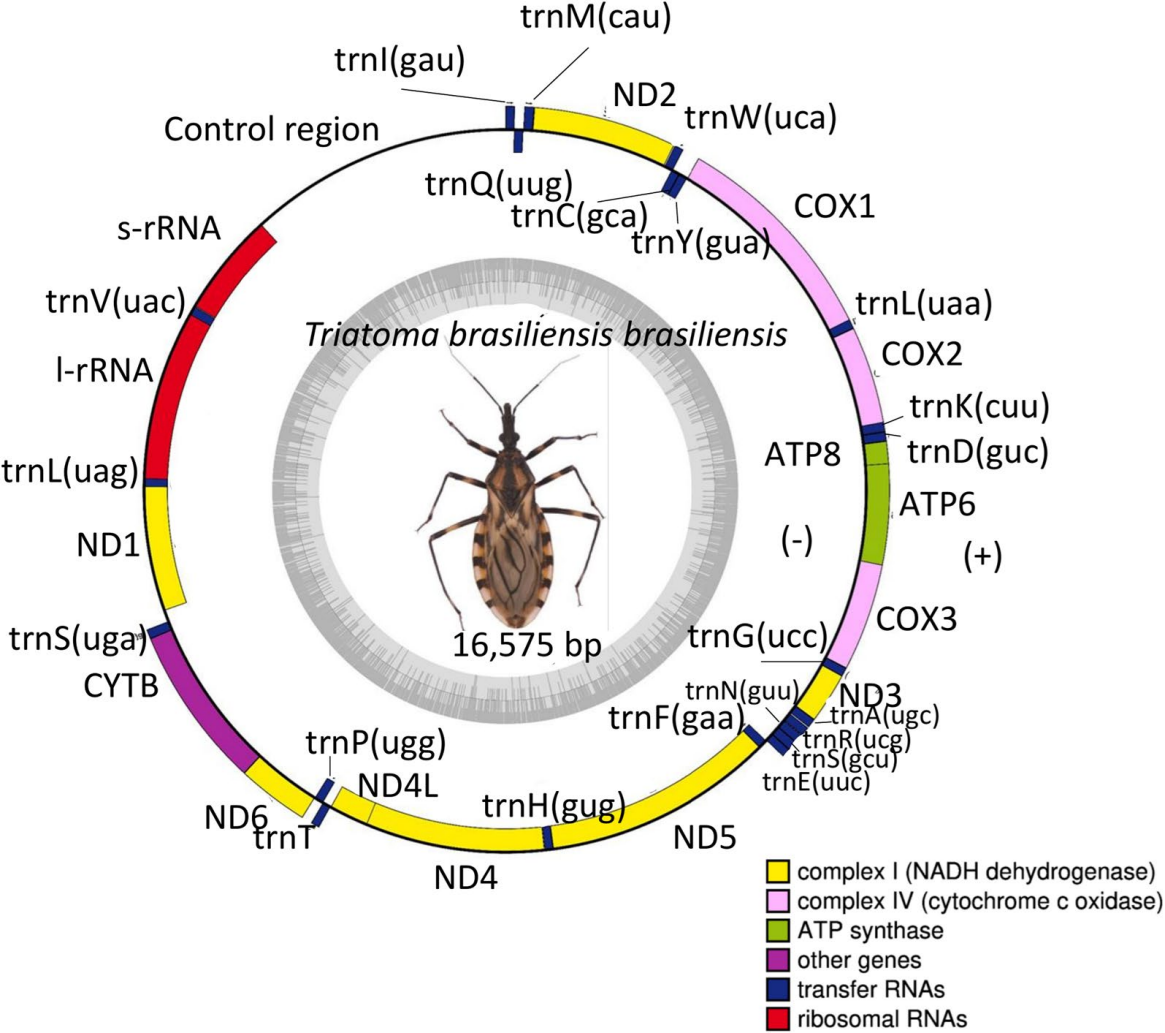
Circular representation of the annotated mitochondrial genome of *Triatoma brasiliensis brasiliensis*. The genome is 16,575 bp long, comprising 37 genes: 13 protein-coding genes (PCGs), 22 transfer RNA (tRNA) genes, and 2 ribosomal RNA (rRNA) genes. The outer ring illustrates the gene arrangement and orientation: genes transcribed on the forward strand are positioned outside the circle, while those transcribed on the reverse strand are positioned outside. Protein-coding genes, tRNAs (represented by their corresponding single-letter amino acid codes), and rRNAs are annotated, with the control region denoted near the origin of replication

A circular map of the *T. b. brasiliensis* mitochondrial genome was constructed (Fig. 1), illustrating the spatial arrangement of all genes, including intergenic regions. The map highlights the genome's structural organization and shows the relative positions of protein-coding genes (PCGs), transfer RNAs (tRNAs), and ribosomal RNAs (rRNAs). The mitochondrial genome of *Triatoma b. brasiliensis* exhibited an A + T-biased codon usage in its protein-coding genes (PCGs), with ATA (isoleucine, 2.95%), ATT (isoleucine, 2.82%), and AAA (lysine, 2.61%) being the most frequently used codons. A complete table detailing codon usage and RSCU (Relative Synonymous Codon Usage) values is provided in Supplementary Information: Additional Table. These results align with codon preferences observed in other heteropteran species, suggesting a conserved pattern in mitochondrial translation efficiency [34] and *T. infestans* [23].

The control region of *T. b. brasiliensis* spans approximately 1500 bp and contains multiple tandem repeats, a characteristic feature of mitochondrial variability in triatomines [35, 36]. This region can be divided into four distinct components, as previously identified [35, 36]: A 149-bp tandem repeat (positions 15,228–15,735) exhibits 100% sequence identity, suggesting a potential structural role in mitochondrial organization. A 120-bp AT-rich repetitive segment (16,007–16,241) has 97% sequence similarity, which may facilitate the secondary structure formation required for mitochondrial replication and species-specific adaptations. A short, structured region within the control region contains an inverted repeat spanning 15,691–16,315, exhibiting 95% sequence identity between its two arms. This inverted repeat (23 bp) has the potential to form a stem-loop structure, a feature commonly associated with mitochondrial replication and gene regulation. Similar to the inverted repeats described in *T. boliviana* [36], this structure may serve as a recognition site for mitochondrial proteins, regulating transcription or replication [35, 36]. A BLASTN search comparing tandem repeat sequences from *T. b. brasiliensis* against mitochondrial genomes of other triatomine species identified a repeat motif with 90% similarity (*E*-value = 4.62e−15, bit score = 68.0) across nine regions in the *T. infestans* (KY640305) control region. No similar matches were found in other triatomine species. Each occurrence of this motif spans 53–57 base pairs, with four mismatches and one gap opening, indicating a reasonable degree of conservation. The presence of recurrent tandem repeats in both *T. b. brasiliensis* and *T. infestans* suggests a potential functional role in the mitochondrial genome, possibly contributing to replication, gene regulation, or structural organization. Furthermore, their distribution across multiple regions in *T. infestans* supports the hypothesis that these sequences may be under selective pressure, maintaining their functional relevance within Triatominae mitochondrial evolution.

**Fig. 2:**
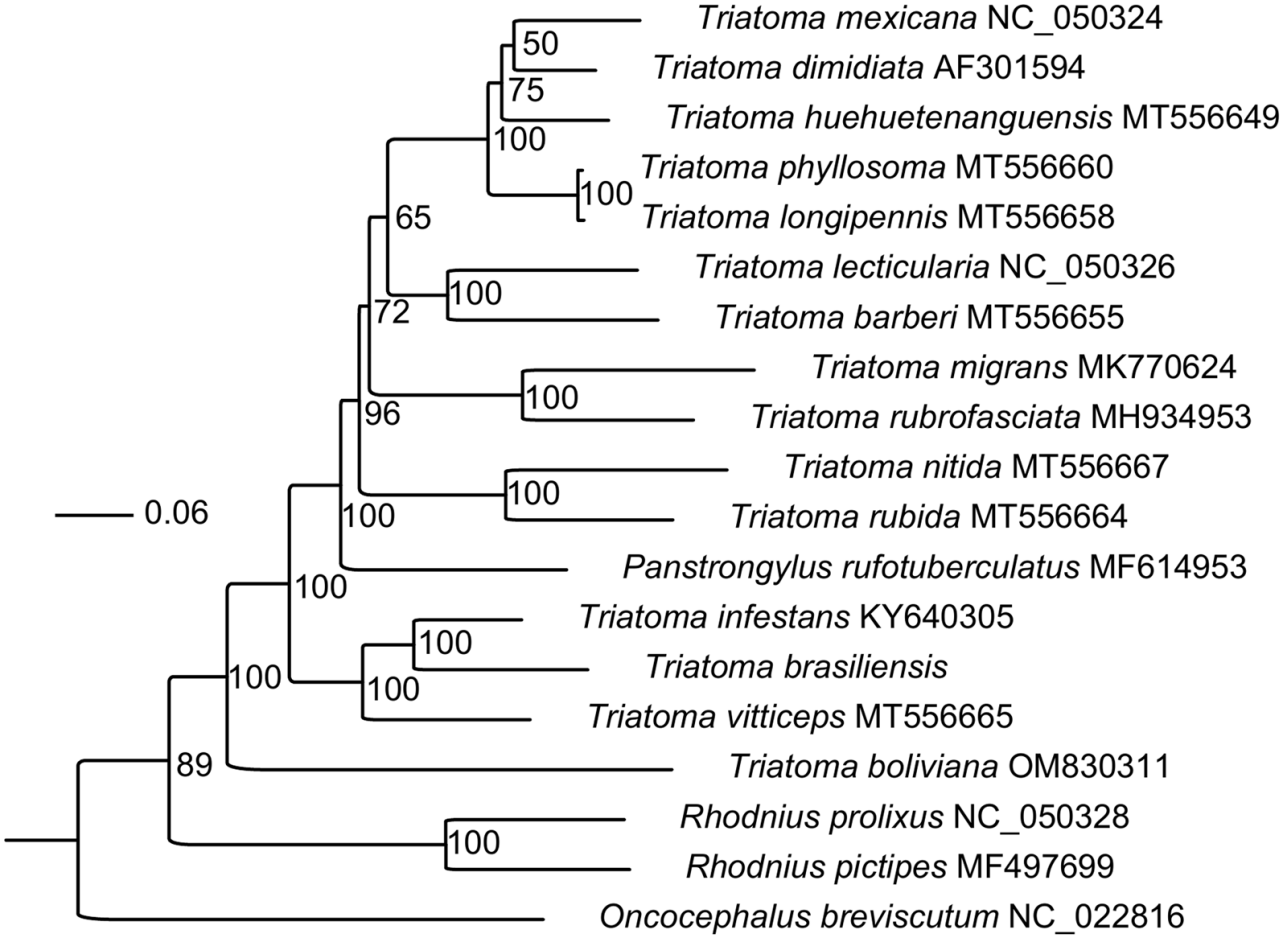
Phylogenetic tree based on conserved blocks of 13 protein-coding genes (PCGs) and 22 transfer RNA (tRNA) genes from representative species complexes with assembled mitogenomes (12,280-bp positions). The tree was constructed using the maximum likelihood (ML) method, with the substitution model TIM2 + F + R4 selected by the Bayesian information criterion (BIC). Support values are indicated at each node. The trace denotes SH-aLRT support

The phylogenetic reconstruction, based on representative species from each species complex with available mitogenomes, did not reveal any significant deviations from the established phylogenies [20, 37, 38]. *Triatoma b. brasiliensis* was strongly supported (BS = 100) as a sister species to *T. infestans*, forming a clade that is sister to *T. vitticeps* (BS = 100). However, the analyzed species represent only a small fraction of the true diversity within the Triatominae. Although Brazil harbors the highest diversity of Triatominae species globally, *T. b. brasiliensis* is only the second endemic species from the country to have its mitogenome annotated (Fig. 2).

## Conclusion

Molecular tools have played a pivotal role in advancing our understanding of the biology and epidemiological impact of *T. b. brasiliensis*. Studies in population genetics [38], molecular ecoepidemiology [11, 39, 40], transcriptomics [41, 42], and other fields have significantly expanded our knowledge of this vector species. These studies have been instrumental in elucidating the adaptive mechanisms, genetic diversity, and epidemiological relevance of *T. b. brasiliensis*. The complete mitochondrial genome of *T. b. brasiliensis* presented here is an addition to the growing genomic resources for Triatominae. Future efforts to sequence and annotate the mitogenomes of other members of the *T. brasiliensis* species complex will be essential for enhancing our understanding of the genetic diversity, ecological adaptations, and phylogenetic relationships within this group of vectors, ultimately contributing to improved management of Chagas disease.

## Supplementary Information

Supplementary Material 1. Codon usage of *Triatoma brasiliensis brasiliensis* mitochondrial genome protein coding genes.

Supplementary Material 2. Complete annotated mitochondrial genome of *Triatoma brasiliensis brasiliensis* (GenBank Accession: PV085522).

## Data Availability

PV085522 (under publication by GenBank in 13-02-25) [a copy was inserted in the end of the manuscript].

## Abbreviations

ATP: Adenosine triphosphate
ATP6: ATP synthase subunit 6
ATP8: ATP synthase subunit 8
BLAST: Basic Local Alignment Search Tool
BS: Bootstrap support
bp: Base pairs
COX1: Cytochrome c oxidase subunit 1
COX2: Cytochrome c oxidase subunit 2
COX3: Cytochrome c oxidase subunit 3
CYTB: Cytochrome b
DNA: Deoxyribonucleic acid
E-value: Expectation value (statistical measure in BLAST)
GC: Guanine-cytosine
Gblocks: Tool for selecting conserved regions in alignments
IQ-TREE: Software for phylogenetic tree construction
ML: Maximum likelihood
ND2: NADH dehydrogenase subunit 2
ND3: NADH dehydrogenase subunit 3
ND4: NADH dehydrogenase subunit 4
ND4L: NADH dehydrogenase subunit 4L
ND5: NADH dehydrogenase subunit 5
ND6: NADH dehydrogenase subunit 6
NCBI: National Center for Biotechnology Information
ORF: Open reading frame
ORFfinder: Open Reading Frame Finder (NCBI tool)
PCGs: Protein-coding genes
RSCU: (Relative Synonymous Codon Usage)
rRNA: Ribosomal RNA
SH-aLRT: Shimodaira-Hasegawa approximate likelihood ratio test
SPAdes: St. Petersburg genome assembler
srRNA: Small ribosomal RNA
TAA, TAG, TTA: Stop codons
*T. b. brasiliensis*: *Triatoma brasiliensis brasiliensis*
tRNA: Transfer RNA
TRs: Tandem repeats
TruSeq: Illumina sequencing library preparation kit
UFBoot2: Ultrafast bootstrap approximation
