# Supplementary material for: The mitogenome of Triatoma brasiliensis brasiliensis (Hemiptera: Reduviidae), the main Chagas disease vector in the semi-arid region of northeastern Brazil
Source: Parasit Vectors. 2025 Apr 4;18:131. doi: 10.1186/s13071-025-06769-0 (PMC11969816; doi:10.1186/s13071-025-06769-0)
Supplement: Supplementary file 1 — Supplementary Material 1. Codon usage of Triatoma brasiliensis brasiliensis mitochondrial genome protein coding genes. [file 13071_2025_6769_MOESM1_ESM.docx]

**Additional File**: Complete Annotated Mitochondrial Genome of *Triatoma brasiliensis brasiliensis* (GenBank Accession: PV085522)

LOCUS PV085522 16575 bp DNA circular INV 12-FEB-2025

DEFINITION topology=circular.

ACCESSION: PV085522

VERSION

KEYWORDS .

SOURCE mitochondrion Triatoma brasiliensis brasiliensis

ORGANISM Triatoma brasiliensis brasiliensis

Eukaryota; Metazoa; Ecdysozoa; Arthropoda; Hexapoda; Insecta;

Pterygota; Neoptera; Paraneoptera; Hemiptera; Heteroptera;

Panheteroptera; Cimicomorpha; Reduviidae; Triatominae; Triatoma.

REFERENCE 1 (bases 1 to 16575)

AUTHORS Almeida,C.E.

TITLE Direct Submission

JOURNAL Submitted (12-FEB-2025) Instituto Oswaldo Cruz, FIOCRUZ, Av. Brasil

4365, Pav. Rocha Lima, Rio de Janeiro, Rio de Janeiro 21040-900,

Brazil

COMMENT Bankit Comment: ALT EMAIL:almeidacaredu@gmail.com

Bankit Comment: TOTAL # OF SEQS:1

##Assembly-Data-START##

Assembly Method :: Mitoz v. 3.6

Sequencing Technology:: Illumina; Sanger dideoxy sequencing

##Assembly-Data-END##

FEATURES Location/Qualifiers

source 1..16575

/organism="Triatoma brasiliensis brasiliensis"

/organelle="mitochondrion"

/mol_type="genomic DNA"

/sub_species="brasiliensis"

/db_xref="taxon:1492235"

gene 1..65

/gene="trnI(gau)"

tRNA 1..65

/gene="trnI(gau)"

/product="tRNA-Ile"

gene complement(63..131)

/gene="trnQ(uug)"

tRNA complement(63..131)

/gene="trnQ(uug)"

/product="tRNA-Gln"

gene 131..198

/gene="trnM(cau)"

tRNA 131..198

/gene="trnM(cau)"

/product="tRNA-Met"

gene 199..1197

/gene="ND2"

CDS 199..1197

/gene="ND2"

/codon_start=1

/transl_table=5

/product="NADH dehydrogenase subunit 2"

/translation="MLNTSTLLFTTTMILGTGIVLSSQEWLGMWMGLEMNLISFIPIL

YKSKNMPSSESCMIYFLIQSLGSFLMLSSVLMNSSIMISPFMGEEFLNTALMLSMMIK

LGMPPCHFWLPEILEKMSWMNCTILMTWQKIAPLCILSHMINSSLLPIIIILSMIAGA

IGGLNQTSLRKIMGYSAIAHMGWMVACMKFNNSFWISYFIIYSMIVIMMTFIFNHYSS

SFINQMINSTPSFMEKSLIITLFMSLGGLPPFIGFMPKWLVIQSMISSSTIIIMLIML

ASSLITLFYYLRIISAILLISSATIKWNQSNGLNPSIVILMITINASLPVISVMNF"

gene 1203..1268

/gene="trnW(uca)"

tRNA 1203..1268

/gene="trnW(uca)"

/product="tRNA-Trp"

gene complement(1261..1323)

/gene="trnC(gca)"

tRNA complement(1261..1323)

/gene="trnC(gca)"

/product="tRNA-Cys"

gene complement(1324..1388)

/gene="trnY(gua)"

tRNA complement(1324..1388)

/gene="trnY(gua)"

/product="tRNA-Tyr"

gene 1390..2928

/gene="COX1"

CDS 1390..2928

/gene="COX1"

/codon_start=1

/transl_table=5

/product="cytochrome c oxidase subunit I"

/translation="MNKWLFSTNHKDIGTLYFMFGAWAGMMGTSLSWLIRIELGQPGS

FIGDDQIYNVVVTAHAFIMIFFMVMPIMIGGFGNWLVPLMIGAPDMAFPRMNNMSFWL

LPPALTLLLMSSLVESGAGTGWTVYPPLSSNIAHSGASVDLAIFSLHLAGVSSILGAV

NFISTIINMRPEGMRPDRIPLFVWSVGITALLLLLSLPVLAGAITMLLTDRNFNTSFF

DPAGGGDPILYQHLFWFFGHPEVYILILPGFGLISHIIAMETGKNEAFGSLGMIYAML

AIGLLGFIVWAHHMFTVGMDVDTRAYFTSATMIIAVPTGIKIFSWLATLHGSVISYSP

SILWALGFVFLFTIGGLTGVILANSSIDIVLHDTYYVVAHFHYVLSMGAVFAIMGGVI

QWYPLFTGMTLNTLWLKIQFMIMFIGVNMTFFPQHFLGLSGMPRRYSDYPDSFVCWNI

ISSLGSTISLIGIIFFLFIIWESMVSKRQVIFPQTLPTNVEWFQKYPPAEHSYSEIPM

ICSS"

gene 2924..2990

/gene="trnL(uaa)"

tRNA 2924..2990

/gene="trnL(uaa)"

/product="tRNA-Leu"

gene 2991..3689

/gene="COX2"

CDS 2991..3689

/gene="COX2"

/codon_start=1

/transl_table=5

/product="cytochrome c oxidase subunit II"

/translation="MATWSNLGTQDANSPLMEQLIFFHDHTLMILTMITILVGYMMST

VLTNKLTNRYLLEGQTIELIWTILPAIILVFIALPSLRILYLMDEVNNPVLTIKSIGH

QWYWSYEYSDFSNVEFDSYMKPINDLETSDFRLLDVDNRVVLPMNSQIRILVTAADVI

HSWTIPSLGIKIDGTPGRLNQGSILINRPGLLFGQCSEICGANHSFMPIVVESVSANQ

FINWLKNNSLSDWK"

gene 3670..3738

/gene="trnK(cuu)"

tRNA 3670..3738

/gene="trnK(cuu)"

/product="tRNA-Lys"

gene 3739..3802

/gene="trnD(guc)"

tRNA 3739..3802

/gene="trnD(guc)"

/product="tRNA-Asp"

gene 3803..3961

/gene="ATP8"

CDS 3803..3961

/gene="ATP8"

/codon_start=1

/transl_table=5

/product="ATP synthase F0 subunit 8"

/translation="MPQMAPSWWTVLFIMFTLSFLIMMFILYSQTYNLPQGIKMNKIT

QPKINWKW"

gene 3955..4660

/gene="ATP6"

CDS 3955..4660

/gene="ATP6"

/note="TAA stop codon is completed by the addition of 3'

Aresidues to the mRNA"

/codon_start=1

/transl_table=5

/product="ATP synthase F0 subunit 6"

/translation="MMTNLFSTFDPATSVNLSLNWLSTFIGFLLIPTTYWMIPSRYNS

LFKTIHLKLYLEFKTLLGPGGTGFSIVFISLFTFILFNNIMGLLPYVFTSSSHLTYTI

TLALPMWLSIMIFGWINHTQHMLAHLIPEGSPSALMPFMVCIETISNIIRPGALAVRL

TANMIAGHLLMSLLGDSVLNSPNYMIPLMMIFQLILVLFETAVSFIQAYVFSMLSTLY

TSEVAYEFTQPPMPPSS"

gene 4625..5409

/gene="COX3"

CDS 4625..5409

/gene="COX3"

/note="TAA stop codon is completed by the addition of 3'

Aresidues to the mRNA"

/codon_start=1

/transl_table=5

/product="cytochrome c oxidase subunit III"

/translation="MSSHNHPYHLVDYSPWPLTGSIGALTLTSGMIMWFHKNDTSLYF

LGILITVLTMIQWWRDITREGTYQGKHTLAVTKGLKLGMILFIISEVFFFISFFWAFF

HSSLAPTVEIGMTWPPKGILTFDPMQIPMLNTMILLCSGITVTWAHHSLMESNHSQAT

QALFITVILGLYFTILQGYEYYESSFTISDSVYGSCFFMATGFHGIHVIIGTTFLAVC

LMRHIMCHFSSKHHFGFEAAAWYWHFVDVVWLFLYISIYWWGS"

gene 5409..5471

/gene="trnG(ucc)"

tRNA 5409..5471

/gene="trnG(ucc)"

/product="tRNA-Gly"

gene 5469..5825

/gene="ND3"

CDS 5469..5825

/gene="ND3"

/codon_start=1

/transl_table=5

/product="NADH dehydrogenase subunit 3"

/translation="MMSKIIISVSTAFFISMMLIIACSVISKKSILDREKMSPFECGF

DPKSSSRMPFSIQFFLIAVLFLIFDIEIVIILPMIITLKTSSLTTWFMTITTFIIILL

IGLYHEWNNGVLEWAN"

gene 5825..5889

/gene="trnA(ugc)"

tRNA 5825..5889

/gene="trnA(ugc)"

/product="tRNA-Ala"

gene 5895..5958

/gene="trnR(ucg)"

tRNA 5895..5958

/gene="trnR(ucg)"

/product="tRNA-Arg"

gene 5960..6024

/gene="trnN(guu)"

tRNA 5960..6024

/gene="trnN(guu)"

/product="tRNA-Asn"

gene 6024..6092

/gene="trnS(gcu)"

tRNA 6024..6092

/gene="trnS(gcu)"

/product="tRNA-Ser"

gene 6093..6155

/gene="trnE(uuc)"

tRNA 6093..6155

/gene="trnE(uuc)"

/product="tRNA-Glu"

gene complement(6158..6225)

/gene="trnF(gaa)"

tRNA complement(6158..6225)

/gene="trnF(gaa)"

/product="tRNA-Phe"

gene complement(6225..7937)

/gene="ND5"

CDS complement(6225..7937)

/gene="ND5"

/codon_start=1

/transl_table=5

/product="NADH dehydrogenase subunit 5"

/translation="MLNKFSVYLLGALALFLSGISSFLVGLFFLVWDYVVFMDWEILS

INSSSITMTFLFDWMSLVFMSCVFIISSMVIYYSESYMGSDVDSVRFYFLVFLFVMSM

MMMIVSPNLMSILIGWDGLGLVSYCLVIYFQNYKSYSAGMLTILSNRVGDVAILLSIA

WMLNFGSWHYIFYMGMWDYWELYLISLIVLAGFTSSAQIPFSSWLPAAMAAPTPVSSL

VHSSTLVTAGVYLLIRFSEMFSNIDCSFILTLSMMTMFMAGLGANFEFDLKKIIALST

LSQLGLMMSILFLGFPILSFLHLLTHAFFKALLFLCAGVMIHCMSDSQDIRHMGVVVN

YLPFTCTCFCISNVSLCGLPFMSGFYSKDLILEAMTSLGYNFFVWLVFFVSVGLTVSY

TFRVIYYVLFSNVNMYVCQSYGEDKVMMKSMIVLALLAIWGGSMLSWLLFYVPELFIF

PFHLKLMPLIFVFLGGWLGYEFSRTNLGEVLLSLRFYFISFFSGSMWFMPYLSTIMLC

GNTLYLSKMYYEAMDGGWGEYIISRSPLLSASLLSKLLNYYQFNNVKIYMAVFALIFI

FSLI"

gene complement(7938..7999)

/gene="trnH(gug)"

tRNA complement(7938..7999)

/gene="trnH(gug)"

/product="tRNA-His"

gene complement(8001..9332)

/gene="ND4"

CDS complement(8001..9332)

/gene="ND4"

/codon_start=1

/transl_table=5

/product="NADH dehydrogenase subunit 4"

/translation="MMSMLAYLIFLIPLCLLNMWWFVVVYMMLGLFYYFHTFWLSDYY

SMVSYSFGGDTLSMCMIFLSFWIIILMIMASYTVYRTGSHSSEFLVVNVVLLFFLVLT

FSTTDLFLFYVFFESSFIPALFLIFGWGYQPERLSAGFYMLFYTLFASLPLLLGIFYI

SSTSGSLFYFLIEVDCNFYLCVSLILAFLVKMPMVFFHFWLPKAHVEAPVAGSMILAG

IMLKLGGYGLLRVSHFIYEYLYAWGYVVIGLSLYGTVLVGVLCLYQIDVKCLIAYSSV

AHMGLVLCGIFSFNYWGFSGALVLMVGHGLCSSGLFCLANIVYERVSSRSLMINKGLI

VFMPSLSLFWFILSSNNMASPPSLNLLGEVMLINGIMSWSPMSSIFLGLSAFLSCCYS

IYLYSYVQHGSIYSGLSKFNFNTFREYALILFHLLPLNFIILKGEIFVLWL"

gene complement(9326..9619)

/gene="ND4L"

CDS complement(9326..9619)

/gene="ND4L"

/codon_start=1

/transl_table=5

/product="NADH dehydrogenase subunit 4L"

/translation="MNLYYYVVFLVMIFSGLLVFCSLRKHLLLTLLSLEFLVLALYFL

FFSFLSMFHLSYYFILVFLTFTVCEGAMGLGVLVSMIRCHGNDNISSLSILGW"

gene 9622..9684

/gene="trnT(ugu)"

tRNA 9622..9684

/gene="trnT(ugu)"

/product="tRNA-Thr"

gene complement(9685..9753)

/gene="trnP(ugg)"

tRNA complement(9685..9753)

/gene="trnP(ugg)"

/product="tRNA-Pro"

gene 9754..10257

/gene="ND6"

CDS 9754..10257

/gene="ND6"

/codon_start=1

/transl_table=5

/product="NADH dehydrogenase subunit 6"

/translation="MMLSTMLLSLTTSITFTMTKHPLSMGLTLIIQTLLVAMMTGMTI

NMFWFSYILTISMLSGMLVLFIYMASVASNEKFHTSWPMTLLIMPLILLSLTLFFIVD

QLETSSMWSTMKKNSVSNEQLISLLKLFNPNNMAITILLVSYLFLTMIAVSYVANSYE

GPLRMKN"

gene 10257..11390

/gene="CYTB"

CDS 10257..11390

/gene="CYTB"

/codon_start=1

/transl_table=5

/product="cytochrome b"

/translation="MNKPLRKTHPLFKIINNSLIDLPAPSSISLWWNFGSLLSMCLMI

QIITGIFLAMHYTGSIELAFSSVIHICRDVNNGWLLRNLHANGASLFFICLYLHIGRG

IYYGSYKLVMTWMMGVIMLFVIMGTAFLGYVLPWGQMSLWGATVITNLLSAIPYLGNE

LVKWLWGGFSVDNATLTRFFALHFLLPFIIAALTMIHLLFLHQTGSNNPLGLTSNFDK

IPFHPYFSTKDLMGVSITLMLFIMLNLWEPRILGDPENFIPANPLVTPVHIQPEWYFL

FAYAILRSIPNKLGGVIAMVSSIAIIMILPLSNKSKFQGLTFYPISQIMFWSLTVILI

LLTWIGARPAEEPYILTGQILTVMYFSYFIINPITLKLWDKIS"

gene 11389..11456

/gene="trnS(uga)"

tRNA 11389..11456

/gene="trnS(uga)"

/product="tRNA-Ser"

gene complement(11540..12475)

/gene="ND1"

CDS complement(11540..12475)

/gene="ND1"

/codon_start=1

/transl_table=5

/product="NADH dehydrogenase subunit 1"

/translation="MVLHVAVFLISYILTLVCILIAVAFTTLMERSVLGYIQLRKGPN

SVGYMGLLQPFSDGLKLFFKEQTYPYYSNFIIYYFSPVFMLMLSFSLWVLFPYMVNVY

NFSFGVLFFLCCTGMGVYGVLLSGWSSNSNYALLGGLRSVAQTISYEVSMALILICML

VFVFSFNFVDFMSYQEYIWFIFFSFPLFFCWLSSCLAETNRSPFDFAEGESELVSGFN

VEYSSGGFAFIFLSEYMNIIFMSLLCCVVFLGCDVYSFMFFIKLTFMVFSFIWVRGTL

PRYRYDKLMYLTWSMFLPLSLNYLIFFSGVILLVV"

gene complement(12458..12522)

/gene="trnL(uag)"

tRNA complement(12458..12522)

/gene="trnL(uag)"

/product="tRNA-Leu"

gene complement(12487..13794)

/gene="l-rRNA"

rRNA complement(12487..13794)

/gene="l-rRNA"

/product="16S ribosomal RNA"

gene complement(13776..13845)

/gene="trnV(uac)"

tRNA complement(13776..13845)

/gene="trnV(uac)"

/product="tRNA-Val"

gene complement(13848..14618)

/gene="s-rRNA"

rRNA complement(13848..14618)

/gene="s-rRNA"

/product="12S ribosomal RNA"

misc_feature 14850..16575

/note="control region"

repeat_region 15395..15735

/rpt_type=tandem

/rpt_unit_range=15395..15543

repeat_region 16163..16283

/rpt_type=tandem

/rpt_unit_range=16163..16283

repeat_region 16216..16273

/rpt_type=tandem

/rpt_unit_range=16216..16273

ORIGIN

BASE COUNT 6647 a 3502 c 1863 g 4563 t

ORIGIN

1 aataaggtgc ctgataaata ggactatttt gatagaatag aatatgtaat aaattaccct

61 tattatattt tttagattta aactaaatcc tgaaagatca aaacttcctg tgcatcatac

121 accaaaatat aaaaagataa gctaataaag ctattaggtt cataccctaa ttatagaagt

181 aaaatcttct tctttttaat cttaaacaca agaacattac tgttcacgac aactataatc

241 ctaggaacag gtattgttct cagatcccaa gaatgattag gaatatgaat aggccttgaa

301 ataaatttaa tttcatttat tccaattctt tacaaatcta agaacatacc atcctcagaa

361 agatgtataa tttactttct aatccaaagt ttaggatcat tcttaatgtt aagatctgta

421 cttataaact cttctattat aatttctccc tttataggag aagagttctt aaatacagcc

481 ctaatactaa gaatgataat taaattagga ataccccctt gccacttctg attgccagaa

541 attttagaaa aaatgtcatg aataaattgc actattctta taacatgaca aaaaattgcc

601 ccattatgca tcctatccca cataattaac agctccctac tacctatcat tattatccta

661 tccataattg caggagcaat cggaggatta aaccaaacat cactacgaaa aattatagga

721 tactcagcaa ttgctcacat aggatgaata gtcgcctgta taaaatttaa taatagattt

781 tgaatcagat acttcattat ttactcaata attgttatta taataacatt cattttcaac

841 cactactcat catccttcat taaccaaata atcaactcaa cgccatcatt tatagaaaaa

901 tccctcatta tcaccttatt tataagacta ggaggacttc ccccctttat cggatttatg

961 cctaaatgat tagtgatcca atcaataatt agatcaagaa caatcatcat catacttatt

1021 atacttgcat cctccctaat caccctattt tattatctac gtattattag agctattctt

1081 cttattagat cagcaacaat taaatgaaat caaagcaatg gactgaaccc aagaattgtt

1141 attctaatga ttacaattaa tgcctcccta cctgtaattt ccgtcataaa cttctagcct

1201 ttaaagcttt aagttaaaaa aactattaac cttcaaagtt aaaattacag gaattaatgt

1261 aagctttagt ataattacta cttcagaatt gcagtctgat atcatcccat tgactataaa

1321 gcctgacaaa gggttactaa ccataaataa atttacaatt taccgcctat tacttcagcc

1381 actttgtcca tgaacaaatg actcttctca acaaaccaca aagatatcgg gactctatat

1441 tttatgttcg gagcctgagc tggaataata ggaacctctc ttagatgatt aattcgaatt

1501 gaactaggac aacccggatc ctttattgga gatgatcaaa tctataacgt tgttgtcaca

1561 gcccatgctt tcattataat tttcttcata gttataccaa tcataatcgg aggattcgga

1621 aactgattag tccccctaat aattggagcc ccagacatag cattcccacg tataaataac

1681 ataagattct gactattacc cccagcctta accttactcc ttataagaag tttggtagaa

1741 agcggagcag ggacaggatg aacagtatac ccgcccttat caagtaatat cgcccacaga

1801 ggagcatctg ttgacctagc aatcttttct cttcacttag caggtgtatc ctccatcctc

1861 ggggctgtaa acttcatttc aactatcatt aatatacgac ctgaaggaat gcggccagac

1921 cgaatcccct tattcgtatg atcagttggc attactgcct tactcctgct tttaagactc

1981 ccagttcttg ctggagcaat taccatatta cttactgacc gaaacttcaa cacttcattc

2041 ttcgacccag ctgggggagg agatcctatt ctttaccaac acttattctg attctttgga

2101 caccctgaag tctacattct aattctccca ggctttggac taatttccca tattattgct

2161 atagaaacag gaaaaaatga agcatttggg tccctaggaa taatttatgc tatactagca

2221 attggcttac taggatttat tgtctgagct caccatatat tcacagtagg aatagacgta

2281 gatacacgag cctacttcac ctcagctaca ataattattg ctgtccctac agggattaag

2341 atcttcagat gactagccac tttacatggt agagtaatct cttacagacc aagaatccta

2401 tgagccctag gattcgtttt cctattcact attggtggcc taactggcgt aatcttagca

2461 aactcaagaa ttgatattgt actacatgat acatactacg tcgtagccca cttccactat

2521 gttctctcaa taggagcagt gtttgctatt atgggaggtg ttattcaatg atacccttta

2581 ttcacaggaa tgacacttaa cactttatga cttaaaatcc aattcatgat catgtttatc

2641 ggagtaaaca taacattctt ccctcaacat ttcctcggat taagaggcat gccccggcga

2701 tactctgatt acccggacag attcgtctgc tgaaacatta tctcttccct aggaagaaca

2761 atctctttaa ttggaattat tttcttccta ttcattattt gagaaagtat agtctccaaa

2821 cgccaagtta tcttccctca aaccctgcct actaacgtag aatgattcca aaagtacccc

2881 ccagcagaac attcatactc agaaatcccc ataatctgtt cttcttaatg tggcagaatt

2941 aagtgcaatg aatttaagct tcatccataa agatccatct ttcattaaga attgctacat

3001 gaagaaatct tggaactcaa gacgctaatt cccccttaat agaacaactc atcttcttcc

3061 acgaccatac ccttataatt ctaacaataa tcactatctt agtagggtac atgataagca

3121 cagtcctcac aaataagctt accaaccgat acctccttga aggtcaaacc attgaattaa

3181 tttgaaccat tttaccagca attatcttag tatttattgc tctccctaga ttacgaattc

3241 tatacttaat agatgaagtt aataatccag tcttaaccat taaatcaatt ggtcatcaat

3301 gatactgaag ttatgaatac tcagacttct caaatgtcga atttgactct tacataaagc

3361 ccattaatga tttagaaaca agagatttcc gtctcctaga cgtagataac cgtgttgttc

3421 tacctataaa tagacaaatc cgtatcctag ttacagccgc agatgtcatc cattcatgaa

3481 ccattcctag actaggaatt aagattgatg gaacacctgg acgacttaat caaggaagaa

3541 ttctaatcaa tcgacctggt ctactattcg ggcaatgctc agaaatctgt ggagcaaatc

3601 atagatttat acctatcgta gtcgaaagag tctctgctaa tcaattcatt aattgactaa

3661 aaaataactc attaagtgac tgaaagtaag taatggtctc ttaaaccaaa atatggtaat

3721 taacatctac ccttaatgaa gaagttagtt taaaaaaaac attagactgt cagactaaaa

3781 atattaatta taatacttct taatcccaca aatagcccca tcttgatgaa cagttctatt

3841 cattatattc actttatcat tccttatcat aatatttatc ctatactcac aaacatataa

3901 cttacctcaa ggcattaaaa taaacaaaat tactcaacca aaaatcaact gaaaatgata

3961 acaaatctat tctccacttt tgacccagcc acttcagtta atttatcact taattgacta

4021 agaacgttca tcggattcct attaattcca acaacatatt gaataattcc atcccgatac

4081 aattcactct tcaaaacaat tcaccttaaa ctttatctag aatttaagac tctcctaggc

4141 cctggaggca caggattttc cattgtattc atttcactct tcacattcat cctatttaat

4201 aacatcatag gcctattacc atacgtattc acaagatcaa gtcacctaac ctacacaatt

4261 actctcgccc taccaatatg actctcaatt ataatctttg gatgaattaa tcacacccaa

4321 cacatgctag cccacttaat cccagaagga agcccctccg ctctcatgcc atttatagta

4381 tgcattgaga ccattagaaa cattattcga cccggagcct tagccgtccg actaaccgct

4441 aatataattg caggccatct cttgatatcc ctattaggag acagagtcct taactcacct

4501 aactacataa ttccattaat aataatcttc caattaattc tagtcctatt tgaaacagcc

4561 gtttcattta tccaagccta cgtattttca atacttagaa cactatacac cagagaagta

4621 gcctatgagt tcacacaacc acccatacca cctagtagat tatagcccat ggcccttaac

4681 aggatcaatt ggagcactaa cattaacatc aggcataatc atatgattcc ataaaaatga

4741 tactagactt tacttcctag gaattctaat cacagttctt acaataattc aatgatgacg

4801 agacattaca cgtgaaggaa catatcaagg gaaacataca cttgctgtca caaaaggatt

4861 gaagttagga ataattttat tcattatttc agaagtcttc ttctttatct cctttttctg

4921 agcattcttc cacagaagat tagccccaac cgtagaaatt ggaataacat gacctcctaa

4981 gggaatccta actttcgacc caatacaaat ccccatatta aatacaataa tcttactttg

5041 ctctggtatt acagtaacat gagcccatca tagactaata gaaagcaacc actcacaagc

5101 tacacaagcc cttttcatta ctgttatctt aggcttatac ttcaccatcc ttcaaggata

5161 cgagtactat gaatctagat tcacaatcag agattccgtt tacggatcat gcttctttat

5221 agcaactgga ttccacggaa tccacgtaat tatcggaaca acattcctag ccgtatgcct

5281 aatacgtcac attatatgtc actttagcag aaagcaccac tttggattcg aagcagcagc

5341 ctgatactga cattttgtag atgtagtctg actattccta tacatctcta tttactgatg

5401 aggtagttat tcctttagta taaaaagtat atttaacttc caattaaaag gtttatcatt

5461 taaaaggaat aatatcaaaa attattatct ctgtatcaac cgcattcttc atttctataa

5521 tacttattat tgcatgctca gtaatctcca aaaagtcaat cctagaccga gaaaaaatat

5581 caccattcga atgcggattc gaccctaaaa gatcatcacg catgcccttc tcaatccaat

5641 tcttcttaat tgcagtcctt ttcttaattt ttgatattga aatcgtcatt atcctaccca

5701 taatcattac attaaaaaca agatccctta caacctgatt cataaccatt acaacattca

5761 tcatcatttt acttattgga ctctatcacg aatgaaataa cggagtactt gaatgagcaa

5821 attaggggct gtagttaaaa ataacatcta atttgcaatt agaaggtgct cactaccgag

5881 ccttcctcac acaaagtagt gaagtaataa ttacatttag tttcgaccta aaaattagag

5941 gtcaacccct ccttacttat taattgaagc caaaacagag gcctttcatt gttaatgaaa

6001 aaattgattt ataatccaat taaaagagaa tgaagaatct aaaagaagct gctaactatc

6061 ttttaaagcg gttaaactcc gtttttctct tcattcatgt agtttaaaga aaacatttca

6121 ttttcaatga aaaaaagaat acacttccat gaattctcac ccagaaagaa tacaccttat

6181 ctaagtatct tcaacacttc gctttcaaat ttaagctatc tgagttaaat caaagaaaaa

6241 ataaaaatta atgcaaagac tgccatataa atcttaacat tattaaactg ataataattt

6301 aataactttc ttaataaaga agcagaaagc aagggcgaac gtgaaataat gtactcaccc

6361 caacctccat ctatcgcctc ataatacatt ttagataaat ataaagtatt cccgcagagc

6421 ataattgttc taagataagg cataaatcat atagaacctc taaaaaaaga aataaaataa

6481 aatcgcaaag aaagcaaaac ctcccctaga ttagttcgag aaaactcata acccagtcaa

6541 ccacccaaaa aaacaaaaat taaaggtatc aacttcaagt gaaagggaaa aataaataac

6601 tcaggaacat aaaaaagcaa ccatcttagt attctgccac ctcaaatagc caaaagagct

6661 aaaacaatta tagacttcat tataacctta tcttccccat aactctgaca aacatatata

6721 ttaacattac taaacaatac ataataaata acacgaaaag tatatgaaac cgtcaaacca

6781 acagaaacaa aaaaaactaa ccacacaaaa aaattataac ccaatctagt tattgcctcc

6841 aaaatcaagt ccttagaata aaaccctgat ataaaaggta acccacataa agacacatta

6901 gaaatacaaa aacaagtaca agtaaaaggt aaataattaa ctaccactcc tatatgacga

6961 atatcctggg aatccctcat acaatgaatc ataacacccg cacataaaaa caacaaagcc

7021 ttaaaaaaag catgcgttaa caaatgcaaa aaagataaaa taggaaaacc aagaaacaaa

7081 atagacatta ttaatcctaa ctgtctcaaa gtagacaaag caataatctt cttcaagtca

7141 aattcaaaat tagcccccaa gccagccata aatatagtca ttatagataa agttaaaata

7201 aaagaacaat caatattaga aaacatctca gaaaaccgaa tcaacaaata aacacccgca

7261 gtaactaaag tagaagaatg aactaaagaa gacacaggag taggagctgc catcgcagca

7321 ggtaatcatg aagaaaaagg aatctgtgct ctcctagtaa acccagctaa aacaatcaaa

7381 gaaataaggt aaagctctca ataatcccac attcccatat aaaaaatata atgccatctt

7441 ccaaaattca acattcaagc aatagacaat aaaatagcaa catctccaac acgattactc

7501 aaaattgtta atataccagc cgagtaagac ttatagttct gaaaataaat aaccagacaa

7561 taagaaacaa ggcctaatcc atcccaacca attaagattc ttattaaatt aggtctaaca

7621 attatcatta ttatagatat aacaaacaaa aaaaccaaaa aataaaaacg aaccctatcg

7681 acatccgagc ccatatatct ctctctataa taaataacta ttgaagaaat aataaaaaca

7741 caagacatga aaactaatga tattcaatca aataaaaaag ttatagtaat agacgatcta

7801 ttaatagaca aaatttccca atccataaaa actacatagt cccaaaccaa gaaaaataaa

7861 ccaactaaaa aagaagaaat accagataaa aataaagcca acgcccctaa aagataaact

7921 gaaaacttat tcaacacgat ctgaagcccc tgggcaccta taattccaca aattataatt

7981 cttcttaaac tattcaaatt ttaaagtcaa agaacaaaaa tctcaccctt taaaataata

8041 aaattcaaag gcaataaatg aaaaagaatc aacgcatact cccgaaaggt attaaaatta

8101 aacttagata aacctctata aatagaacca tgctgaacat aagaatataa ataaatgcta

8161 taacagcatc ttaaaaaagc agaaagcccc aaaaaaatag aacttatagg actccatctc

8221 ataattccat taattagcat cacttcacct aacaaattca aggaaggcgg agaagccata

8281 ttattggaac ttaaaataaa ccaaaacaaa gaaagagaag gcataaaaac aattaaaccc

8341 ttattaatca taagtctccg actagaaaca cgctcataaa caatattagc caaacagaaa

8401 agaccagaag aacacaaacc atggccaacc atcaaaacca aagcacccga aaaccctcaa

8461 taattaaaag aaaaaatccc acacaagacc aaacctatat gagccacaga agaataagca

8521 attaaacact tcacatcaat ttgatacaaa cataaaaccc caactaacac agtaccatac

8581 aatctcaagc caattacaac ataccctcaa gcatataaat attcataaat aaaatgagaa

8641 acacgtaaaa gaccataccc ccctaacttc aacataatcc ccgccaaaat catagaaccg

8701 gcaacaggag cttccacatg agccttaggg agtcaaaaat gaaagaaaac tataggcatc

8761 ttaaccaaaa aagctaaaat taaagagaca cataaataaa aattacaatc aacctcaatt

8821 aaaaaataaa ataatctacc agaagtccta ctaatataaa aaatacccaa caacaaaggc

8881 aaagaagcaa acaaagtata aaataatata taaaaccctg ctcttaaacg ctcaggctga

8941 tagcctcaac caaaaatcaa aaacaaggca ggaataaaag aagactcaaa aaatacataa

9001 aacaaaaaga gatcagtagt agaaaaagta agaaccaaaa aaaataataa aacaacatta

9061 accacaagaa attcacttct atggctaccc gtgcgataca cagtatatct agctataatc

9121 ataagaataa taatccaaaa tctcaaaaag atcatacata tagacaaagt atcccctcca

9181 aaagagtatc taaccatact ataataatca gaaagtcaaa aagtatgaaa ataataaaat

9241 aaacctaata tcatataaac aacaacaaac caccacatat tcaataaaca aagagggatc

9301 aaaaaaatta aataagccag tattcttatc atcccaaaat agaaagtcta gaaatattgt

9361 cattaccatg acaacgaatt attctcacta aaacacctaa acccatagcc ccctcacata

9421 cagtaaaagt taaaaaaacc aagataaagt aataggacaa atgaaacata gaaagaaaag

9481 aaaaaaacaa aaaataaaga gccaacacca aaaactcaag tcttaaaaga gttaaaagta

9541 aatgtttacg caaagaacaa aaaacaagta aaccagaaaa aatcatgacc aaaaaaacta

9601 cataataata taaattcatc agttttaata gtttaaaaaa aataatgatc ttgtaaatca

9661 tagatagaaa taatctttaa aacttcagca agaagagaat aacacctccc atcattaatc

9721 cccaaaatta atatttttat aaactactta ctgataatgc tatcaactat acttctctcc

9781 ctaactacaa gaattacatt tacaataacc aaacaccccc taagaatagg attaacctta

9841 attattcaaa ccctcctagt agcaataata acaggcataa ccattaacat attctgattc

9901 tcatacatcc taacaatctc aatattaaga ggtatgctag ttttattcat ctacatagca

9961 agagtggcat caaacgaaaa atttcacaca tcctgaccca taaccttact tattataccc

10021 ttaattcttc tatcccttac cctattcttt attgtggatc aattagaaac cagaagaata

10081 tgatccacaa taaaaaagaa tagagtaaga aatgaacaac ttatttcact tcttaagtta

10141 ttcaacccaa ataacatagc aattactatt ctcctcgtct cctacctctt cctaacaata

10201 attgcagtct cctacgtagc taactcatac gaaggacctt tacgcataaa aaactaatga

10261 acaagccatt acgcaaaacc caccccctat tcaaaatcat taacaattca ctaattgacc

10321 taccggcccc ctcaagaatc tccttatgat gaaattttgg gtccctccta agaatatgcc

10381 taataattca aattatcaca ggaatcttct tagcaataca ttacacagga agaattgaac

10441 tagcattcag aagagtaatc catatttgcc gtgatgttaa caacggatga ttacttcgca

10501 acctccatgc taatggcgca tcattatttt tcatttgcct ctacctacat atcggacgag

10561 gcatttatta tggatcctac aaattagtca taacctgaat gataggtgtt atcatactgt

10621 ttgtgattat aggcactgcc ttcctaggat acgtactacc ctgaggtcaa atatccctgt

10681 gaggagctac agttattact aacctcttat ctgccatccc ctacctaggt aacgaactcg

10741 taaaatgact atgaggagga ttctccgttg acaatgctac tttaacacga ttctttgccc

10801 ttcacttcct cctaccattt attatcgctg ccctcacaat aatccatcta ctattcctac

10861 accaaacagg atctaataat ccattaggat taacaagaaa cttcgacaaa atcccattcc

10921 atccctattt ctcaactaag gatctaatag gagtatcaat tacattaata ctatttatta

10981 tacttaactt atgggaacca cgaattttag gagatccaga aaattttatc ccagcaaacc

11041 cattagtaac tccagttcac attcaacctg aatgatactt cttattcgca tatgccattc

11101 tacgatcaat tccaaataaa ctcggaggag ttattgccat agtatcatca attgcaatca

11161 ttataatcct ccctctctct aacaaaagaa aattccaagg actaacattt tacccaatca

11221 gccaaatcat attctgatca ctaactgtta ttttaatcct cctaacatga attggagctc

11281 gaccagcaga agaaccctac attttaacag gccaaatctt aactgttata tacttctcct

11341 atttcattat taacccaatc actttaaagc tatgagacaa aatctcctag ttaattagct

11401 taaagaaagc ctgtattttg aaagtacaag aaaaaggtta atccctttat taacttcact

11461 tttttaaatc attagttctc taaaaaaagt tctcccctca atatttcatt aatttaattc

11521 actaaaaaga atgctaaaat tatacaacta acagaataac tcctgaaaag aaaattaagt

11581 aattaagaga caaaggcaaa aatatccttc aagttaaata catcaactta tcataacgat

11641 aacgaggcaa agttccccga actcaaataa aagaaaaaac cataaaagtc aatttaataa

11701 aaaatataaa tgaatacaca tcacaaccca aaaaaaccac acaacataaa agtcttataa

11761 aaataatatt tatatactca gacaaaaaaa taaaagcaaa cccccctctt ctatactcaa

11821 cattaaaacc agaaaccaac tctgactccc cttcagcaaa atcaaaggga gaacgattag

11881 tttctgccaa acaagaagaa agccaacaga aaaacaaagg aaaagaaaaa aaaataaatc

11941 aaatatactc ctgatacctc ataaaatcaa caaaattaaa tctaaaaaca aaaactaaca

12001 tacaaattaa aattaaagcc attcttactt cataagaaat agtctgagcc actgaacgta

12061 acccacccaa aagagcataa ttagaattag atctccaccc agacaataag actccataaa

12121 cccctatacc cgtacaacaa agaaaaaaca aaactccaaa actaaaatta taaacattta

12181 ctatataagg aaacaaaacc cacaacgaaa atgacaacat taacataaaa acaggagaaa

12241 aataataaat aataaaatta gaataataag gataagtttg ctccttaaaa aataatttta

12301 acccatcaga aaaaggctga agcaaaccta tataccccac cctattagga cccttacgta

12361 actgaatata gcccaaaacc cttcgctcca tcaaagttgt aaaagcaaca gcaatcaaaa

12421 tacaaaccaa agtcaaaata taagaaatca aaaacactgc tacatgtaga acaatctaca

12481 taaataaatt ctaagtttat tgcactaatc tgccaaaata gcaatataaa tcaaatcaaa

12541 aaaaattatt ccatacattt ggtcctttcg tactaaaatg tataacaaat ttaaagatag

12601 aaactgacct ggctcacgcc ggtctgaact cagatcatgt aaaaatttaa aggtcgaaca

12661 gacctagtaa atagactcct gcatccatga cttattttaa tccaacatcg aggtcgcaaa

12721 ctccctcatc aatatgaact ctccgaggaa attacgctgt tatccctaag gtaacttaat

12781 cttataatca taaactatgg atcaaaaaaa cactaattaa tgaaaataaa aaatagaagt

12841 tattcaaatt ctacagtcac cccaacaaaa tagttaacct acttaaagta aataaatccc

12901 taaattacca aaatataaaa ctataaagat ctatagggtc ttctcgtccc ataaataaat

12961 ttaagctttt taactcaaaa attaaattct taaaattaaa ttaaagaaag tcaatacctc

13021 gtccaaccct tcattccagc cctcaattaa aagacaaatg attatgctac cttagcacag

13081 ttagaatact gcggccattc aattcctcat tgggcaggtc agaccttaaa taaacaaaca

13141 aaaggacatg tttttgttaa acaggcgggc actaaaattg ccgagttcct ataatttaat

13201 tctctaaaat actaattcaa tcattattac aacaaattac aaattaaatt aatcattcaa

13261 gtaaataaat aaagttaaat aaataaacta cataaagaaa taatctataa cgaaataaat

13321 gatggctgat ttcaagccta cgaaattcct aaaaacatca caacttatat aattaatcct

13381 gagcttatcc ccaagaatat tagaacttca tagtaaaaaa aataaaagta tccattaccc

13441 taataaaaat cctaaattaa atttaattcc aagaaaacca gatattcaag aacgaataac

13501 atttcattac cagaactaaa taatcaataa ttttgacaca ttaaataaca cttagtccta

13561 gctctcctaa tttcgggaaa tatatataaa tataataatt taattgaccc tgatacaaaa

13621 ggtacgccaa ataataacta cttataaaca atataatatt aatccttcac agtactataa

13681 actataatca aaataatttt ttcaataaaa tatactaaaa acgaagttac ttctaaaaaa

13741 aaataaacac taaaaaaaat aataaaaatt aaacttcaaa tcaagttgaa tcgcacaact

13801 aaactattaa tgtaaataat aatacctcct tgagattatg atttgataat ataccagatt

13861 caccttccag taaatctact ttgttacgac ttatctcatt ttaaataaac gagagtgacg

13921 ggcgatatgt gcataattta gagctaaaat caaatctaca ttctaataga aattacttcc

13981 aaatccaatt tcaaaaatat ttccatattc actatccata aatacattta atgtaatcca

14041 cctcttctct accattgctg caccttgacc tgacattttg cacattaaag attccagaaa

14101 ataccctaat aggacattca atgacagaga tatacaagcc caggcaaagt aaaattcatc

14161 gtggaatatc aattacaggg cagattcctc tgaacagact aaattaccgc caaattcttt

14221 taatttaaag accataacta ttaatactaa gattaccaaa ttactgttta tgataatggg

14281 gtctctaatc ccagtttcac attaaacttt catattttaa tcctaccaat tcatataata

14341 aaaaatctaa atttcaccac aagatcattc attaaaatga aataaataaa aataaataca

14401 aaccaaactc aattcactca cccctattgt ataaccgcga ctgctggcac aacattggtc

14461 agaattcttt taattaacta aatctacatt tctgtaatat ataaaattaa gaactgagaa

14521 tcaaatttcc aaatcatcta gaaacccacc tgatcccaca aaaatataca tgtacaatca

14581 ataaaaagca aatttaataa gccagaatca aactttaata aaaagaaatt atacttggta

14641 cattatcaaa tgtatccccc cctcctcttg acctcccctg aagccctgac ccgaaagtgc

14701 gacgttcaag attttttact ataatatagg aatactagat attgacatat tattaaatat

14761 tttatatgat taaattatca cataactgta aatggttgta atggtccatt acgatataac

14821 ttccattagc gatcccagtt agaattgacg ctcagaggta acaccccttc atttctactt

14881 caggtagaaa tgacggctgg ctctgcccgt acagcaaacg tgtactataa taccctatca

14941 taagtttcca cggcaggaaa atatttacat acctatacct ggatcctttc ccatagattc

15001 ctctacttcc catatgtccc atagactatg tcctaccatg tctccctaaa tagtttcata

15061 cccggatagg attagggggg gggggggtct tacatacaaa tttatatttt tacaatataa

15121 atgcattcca tgcaagctaa aagcttacat atccctttaa agcatattag ttgttaacta

15181 ataacatatg attaaaaatc atacttctat gctttactat taaaattaaa atcaaaaatt

15241 aaaattatta ttatatatga aactaaccaa ctctagcacc tacaattcag aatttgtccc

15301 ttcaaataaa tcaaaatcaa aactatacgc gatcttcaaa ataatccaag attccaattc

15361 ctcaaaataa acgctcaaaa tcaaaaatta aaattattat tatatatgaa actaaccaac

15421 tctagcacct acaattcaga atttgtccct tcaaataaat caaaatcaaa actatacgcg

15481 atcttcaaaa taatccaaga ttccaattcc tcaaaataaa cgctcaaaat caaaaattaa

15541 aattattatt atatatgaaa ctaaccaact ctagcaccta caattcagaa tttgtccctt

15601 caaataaatc aaaatcaaaa ctatacgcga tcttcaaaat aatccaagat tccaattcct

15661 caaaataaac gctcaaaatc aaaaattaaa attattatta tatatgaaac taaccaactc

15721 tagcacctac aattccaaaa tatacacgcc cctacaaaac cgcatgttca gaaaatctga

15781 acacgaacaa aaacaaatat tacactaacc cttcaacgac aaagtctggc aatattacga

15841 ttcaagatat acacgccctt tcacaatcgc ctgttcagaa atctgcacac ggactaaatc

15901 aggtatttca ctatccttct atgacaagcc ggcaaaatgc gtggtgcgca aatccgaacc

15961 agatgtacct caattttctc atttgccctt taatgtctta tgcaccaaga ataaacctgg

16021 caatctaaca ataaaaatat acacgcccct acaaaaccgc atgttcagaa aatctgaacc

16081 cgaactaaaa caaatattac actaaccctt caacgacaaa gccaaaaaga ataaatctgg

16141 caatctaaca ataaaaatat acacgcccct acaaaaccgc atgttcagaa aatctgaaca

16201 cgaacaaaaa caaatattac actaaccctt caacgacaaa gaaggcaaaa ataagatgag

16261 cacaaaacca aaacaaaagt accaaaatat tataatttca tatataatat ataatacccc

16321 aaaaataaac ctggcaattt aacaataaaa atatacacgc ccctacaaaa ccgcatgttc

16381 agaaaatttg aacacgaaca aaaacaaata ttacactaac ccttttcatg gcaaaggaag

16441 caagacataa aacaaaaaat acaaaataaa taaagccaag ccccaataac aaaattatac

16501 ccattaaaga ttcaaagacc atacaaaatc aacgatcccc gttttaaaag ggaaaaaaaa

16561 acggggatta taaat

//
