# Supplementary material for: The mitogenome of Triatoma brasiliensis brasiliensis (Hemiptera: Reduviidae), the main Chagas disease vector in the semi-arid region of northeastern Brazil
Source: Parasit Vectors. 2025 Apr 4;18:131. doi: 10.1186/s13071-025-06769-0 (PMC11969816; doi:10.1186/s13071-025-06769-0)
Supplement: Supplementary file 2 — Supplementary Material 2. Complete annotated mitochondrial genome of Triatoma brasiliensis brasiliensis (GenBank Accession: PV085522). [file 13071_2025_6769_MOESM2_ESM.docx]

**Additional Table**: Codon usage of *Triatoma brasiliensis brasiliensis* mitochondrial genome protein coding genes.

| Codon | Amino Acid | n | % | RSCU |
| --- | --- | --- | --- | --- |
| ATC | I | 124 | 1.67 | 0.67 |
| TTA | L | 98 | 1.32 | 1.64 |
| AAC | N | 134 | 1.81 | 0.96 |
| ACA | T | 118 | 1.59 | 1.84 |
| AGA | R | 149 | 2.01 | 3.84 |
| CTG | L | 18 | 0.24 | 0.3 |
| TTC | F | 122 | 1.64 | 1.39 |
| ACG | T | 18 | 0.24 | 0.28 |
| ACT | T | 46 | 0.62 | 0.72 |
| ATA | I | 219 | 2.95 | 1.19 |
| CTA | L | 103 | 1.39 | 1.72 |
| GGA | G | 98 | 1.32 | 2.67 |
| GGT | G | 15 | 0.2 | 0.41 |
| ATT | I | 209 | 2.82 | 1.14 |
| GTT | V | 33 | 0.44 | 1.11 |
| CTC | L | 63 | 0.85 | 1.05 |
| TCC | S | 55 | 0.74 | 1.11 |
| CAA | Q | 147 | 1.98 | 1.95 |
| GAA | E | 58 | 0.78 | 1.78 |
| TGA | W | 81 | 1.09 | 1.37 |
| GGC | G | 21 | 0.28 | 0.57 |
| CTT | L | 71 | 0.96 | 1.19 |
| AAT | N | 144 | 1.94 | 1.04 |
| TCA | S | 87 | 1.17 | 1.76 |
| TTT | F | 54 | 0.73 | 0.61 |
| CCA | P | 66 | 0.89 | 1.59 |
| TAC | Y | 65 | 0.88 | 1.05 |
| AAA | K | 194 | 2.61 | 1.57 |
| TCT | S | 60 | 0.81 | 1.22 |
| AAG | K | 53 | 0.71 | 0.43 |
| TGT | C | 8 | 0.11 | 0.53 |
| AGT | S | 45 | 0.61 | 0.91 |
| ATG | M | 42 | 0.57 | 1.0 |
| GTA | V | 49 | 0.66 | 1.65 |
| CCC | P | 57 | 0.77 | 1.37 |
| GAG | E | 7 | 0.09 | 0.22 |
| GCC | A | 44 | 0.59 | 1.5 |
| CCT | P | 36 | 0.48 | 0.87 |
| TGC | C | 22 | 0.3 | 1.47 |
| CAC | H | 55 | 0.74 | 0.89 |
| TTG | L | 6 | 0.08 | 0.1 |
| AGC | S | 46 | 0.62 | 0.93 |
| GCA | A | 43 | 0.58 | 1.47 |
| CGA | R | 27 | 0.36 | 0.7 |
| GCT | A | 29 | 0.39 | 0.99 |
| GTC | V | 31 | 0.42 | 1.04 |
| ACC | T | 75 | 1.01 | 1.17 |
| GTG | V | 6 | 0.08 | 0.2 |
| TAT | Y | 59 | 0.79 | 0.95 |
| CGT | R | 12 | 0.16 | 0.31 |
| TAG | * | 7 | 0.09 | 0.12 |
| GAT | D | 27 | 0.36 | 0.96 |
| GGG | G | 13 | 0.18 | 0.35 |
| CAT | H | 69 | 0.93 | 1.11 |
| GAC | D | 29 | 0.39 | 1.04 |
| CCG | P | 7 | 0.09 | 0.17 |
| CGG | R | 3 | 0.04 | 0.08 |
| CGC | R | 12 | 0.16 | 0.31 |
| TAA | * | 89 | 1.2 | 1.51 |
| TGG | W | 2 | 0.03 | 1.0 |
| AGG | R | 30 | 0.4 | 0.77 |
| TCG | S | 3 | 0.04 | 0.06 |
| CAG | Q | 4 | 0.05 | 0.05 |
| GCG | A | 1 | 0.01 | 0.03 |
